# Characterization and Behavioral Responses of Volatile Organic Compounds in Commercial Cockroach Baits

**DOI:** 10.3390/insects17101010

**Published:** 2026-09-30

**Authors:** Kejia Chen, Bin Sun, Hongyan Lyu, Xinhong Xu, Lu Jiang, Xiaomeng Ren, Huiling Hao, Yunchuan He

**Affiliations:** Chinese People’s Liberation Army Naval Medical Center, Shanghai 200433, China

**Keywords:** *Blattella germanica*, volatile organic compounds (VOCs), cockroach bait, attractant screening

## Abstract

Cockroach baits are widely used due to their high efficacy and low environmental impact. However, a systematic understanding of the volatile organic compounds (VOCs) that mediate attraction behavior in *Blattella germanica*, and their underlying mechanisms is still lacking. In this study, the volatile profiles of 15 commercially available baits were systematically characterized using headspace gas chromatography–mass spectrometry (HS-GC-MS). The behavioral and electrophysiological responses of *B. germanica* to 14 candidate VOCs were further evaluated using a Y-tube olfactometer and electroantennography (EAG). The results revealed significant differences in VOC composition among the tested baits, with aldehydes, acids, alcohols, and esters constituting the major components. This study identifies 2-methyl-3-buten-2-ol and hexanal as the key bioactive compounds responsible for attracting *B. germanica* in commercial baits. Their strong attractiveness at low concentrations indicates substantial practical application potential. These findings not only elucidate the chemical basis of the attractant activity of cockroach baits but also provide critical theoretical insights and technical support for optimizing existing bait formulations and developing novel, highly effective attractants.

## Simple Summary

Cockroaches are common household pests that contaminate food, damage household materials, and transmit pathogenic microorganisms. Baits are widely adopted for cockroach management owing to their high efficacy and low environmental risk. However, the specific volatile compounds mediating cockroach attraction to baits remain largely uncharacterized. In this study, we analyzed the chemical profiles of 15 commercial cockroach baits using headspace gas chromatography–mass spectrometry (HS-GC-MS). We further investigated olfactory responses of *Blattella germanica* to 14 selected compounds via Y-tube olfactometer and electroantennography (EAG) assays. Our results revealed distinct volatile profiles among tested baits, with aldehydes representing the predominant chemical class. Two compounds, 2-methyl-3-buten-2-ol and hexanal, elicited attractive effects on *B. germanica* at low concentrations. These findings advance our understanding of the chemical basis underlying bait attraction and offer valuable information for developing more potent cockroach control formulations.

## 1. Introduction

Cockroaches are serious sanitation pests in human habitats worldwide. They contaminate food, damage property, and transmit various pathogenic microorganisms, posing a serious threat to human health and quality of life [1,2,3]. Chemical control plays a central role in integrated cockroach management, with residual spraying and baits being the most widely used methods. However, the long-term and extensive use of spray insecticides has led to increasingly serious resistance problems in cockroach populations, and their associated societal costs, lack of observable benefits, and the availability of highly efficacious, affordable alternatives further limit the sustainability of this approach [4,5,6]. In contrast, cockroach baits have gradually become the mainstream technology for indoor cockroach control due to their high safety, long residual activity, low insecticide dosage, and minimal environmental residues [4,5,6]. The efficacy of baits largely depends on their attractant and feeding stimulant properties, with volatile organic compounds (VOCs) serving as key long-range olfactory signals that play a decisive role in guiding cockroaches to locate, approach, and consume the baits [7].

The German cockroach (*Blattella germanica*) relies primarily on its highly sensitive olfactory system to detect volatile chemical cues in the environment, thereby completing behavioral responses such as food seeking, aggregation, and mate location [8,9]. Some sex pheromones and aggregation pheromones, can exhibit attractive effects on specific cockroach species, However, their large-scale application in commercial baits is limited by unpleasant odors, high synthesis costs, or environmental sensitivity [10,11]. Currently, most commercially available cockroach baits rely primarily on food-derived attractants (e.g., sugars, proteins, and starches) as their basic attractant matrix [12,13], but their key active volatile components remain unclear, and the underlying mechanisms of olfactory recognition and behavioral attraction are still lacking systematic understanding.

Therefore, this study comprehensively analyzed the volatile profiles and validated the behavioral activity of 15 commercially available cockroach baits using HS-GC-MS, electroantennography (EAG), and Y-tube olfactometer assays. The aim was to screen and validate the key VOCs responsible for attracting *B. germanica*, thereby identifying the core components among them, and providing a chemoecological basis for the optimization and development of highly attractive cockroach baits.

## 2. Materials and Methods

### 2.1. Materials

Insects: The *B. germanica* strain used in this study originated from wild populations collected in Shanghai. After long-term multi-generational rearing under standardized laboratory conditions, this field population was domesticated into a genetically stable susceptible strain and has since been continuously maintained under controlled environmental conditions. The cockroaches were reared in an artificial climate chamber (TH-PACHB-C1006-T1, Shanghai Shibei Instrument Equipment Factory, Shanghai, China) with a 12 L:12 D photoperiod at 26 ± 0.1 °C and 60% relative humidity. Adult females and males (7–14 days post-emergence) were selected for EAG and olfactory behavioral assays. All tested adults were unmated and age-synchronized. Prior to olfactory behavioral assays, the test insects were starved for 24 h with access to water.

Cockroach baits: Fifteen commercially available cockroach baits, which are widely sold and used in mainland China, were selected for this study. The insecticidal active ingredients included indoxacarb, imidacloprid, dinotefuran, hydramethylnon, and cockroach virus. Further information on the baits is provided in Table S1.

Reagents: 2-methyl-3-buten-2-ol (CAS: 115-18-4, 98%); 2-ethyl-1-hexanol (CAS: 104-76-7, 99%); hexanal (CAS: 66-25-1, 97%); ethanol (CAS: 64-17-5, 99.5%); acetic acid (CAS: 64-19-7, 80%); propanoic acid (CAS: 79-09-4, 99.5%); 2-methylfuran (CAS: 534-22-5, 98%); sorbic acid (CAS: 110-44-1, 99.8%); propylene glycol (CAS: 57-55-6, 99%); ethyl butanoate (CAS: 105-54-4, 99%); p-anisaldehyde (CAS: 123-11-5, 99%); γ-nonalactone (CAS: 104-61-0, 98%) and GC-MS grade methanol (CAS: 67-56-1, 99.9%). These reagents were purchased from Macklin Biochemical Technology Co., Ltd., Shanghai, China. 3-methyl-1,2-cyclopentanedione (CAS: 765-70-8, 98%) and D-limonene (CAS: 138-86-3, 95%) were purchased from Aladdin Biochemical Technology Co., Ltd., Shanghai, China.

### 2.2. HS-GC-MS Analysis

The separation and identification of volatile compounds were performed using a HS-GC-MS system (Agilent Technologies Inc., Santa Clara, CA, USA). The system consisted of an Agilent 7697A headspace sampler, an Agilent 8890 GC system, and an Agilent 5977B GC/MSD mass spectrometry detector (Agilent Technologies Inc., Santa Clara, CA, USA). Cockroach bait samples (0.85 ± 0.05 g) were placed into 20 mL headspace vials (20 mL, 22.5 × 75 mm, clear glass, crimp top, flat bottom) and equilibrated at room temperature for 60 min before analysis. Four replicates were performed for each bait.

The HS-GC-MS conditions were as follows: The carrier gas was high-purity helium at a constant flow rate of 1.0 mL/min. Headspace conditions: equilibration at 60 °C for 20 min; injector temperature 250 °C with a split ratio of 5:1. The separation was performed on an Agilent DB-624 UI column (length 60 m × inner diameter 0.25 mm × film thickness 1.4 μm, part number 122-1364UI, Agilent Technologies Inc., Santa Clara, CA, USA). The oven temperature program was as follows: 40 °C for 5 min, increased to 150 °C at 10 °C/min and held for 7 min, then increased to 240 °C at 20 °C/min and held for 10 min. The mass spectrometer was operated in electron ionization (EI) mode with an ion source temperature of 230 °C, and mass spectra were recorded over the range of *m*/*z* 35–300.

Compound identification was performed by matching mass spectra against the NIST 20 mass spectral library, with the highest matching score above 80 used as the basis for qualitative identification, match scores fall below 80, rendering them unreliable for qualitative analysis. Based on the HS-GC-MS results, detection frequency, peak intensity and article reported [14,15,16,17,18,19,20,21,22,23,24,25,26,27,28,29,30], 14 compounds were selected for EAG and Y-tube olfactometer assays. These compounds were further confirmed by standards (Figure S1).

### 2.3. EAG Recording

The EAG system used in this study was manufactured by Ockenfels Syntech GmbH, Kirchzarten, Germany. The setup consisted of a stimulus controller (CS-55; Ockenfels Syntech GmbH, Kirchzarten, Germany), a micromanipulator (MP-15; Ockenfels Syntech GmbH, Kirchzarten, Germany), the PRG-3 EAG Kombi electrode (Ockenfels Syntech GmbH, Kirchzarten, Germany), a two-pronged fork-shaped probe specifically intended for electrophysiological recording from excised filiform antennae and a data acquisition controller (IDAC-2; Ockenfels Syntech GmbH, Kirchzarten, Germany).

To prepare the antenna, the antenna of living adult *B. germanica* (both male and female) was quickly excised from the base using a surgical blade, and the tip (approximately 0.1 mm) was cut off. The prepared antenna was then placed between two glass capillaries filled with 0.9% saline solution, which were mounted on the PRG-3 electrode. For stimulus preparation, 20 μL of each test compound (neat) was applied to a cotton wick (length × width = 20 mm × 5 mm). The wick was placed inside a 1000 μL pipette tip, which was then connected to the tubing of the stimulus controller (CS-55; Ockenfels Syntech GmbH, Kirchzarten, Germany). Recordings were performed after the baseline signal had stabilized. The continuous airflow was set at 9 mL/s, while the pulsed stimulus airflow was set at 5 mL/s. Each stimulus pulse lasted 0.3 s, with an interval of 30 s between consecutive stimuli. When switching between different test compounds, an additional recovery period of at least 1 min was allowed to ensure the antennal signal fully returned to baseline before the next compound was introduced. For each adult, only one antenna was used. The antenna sequentially received all test compounds (three stimulations per compound). The order of compound presentation was randomized to avoid errors caused by antennal inactivation, and five biological replicates were performed for each sex. The response value was recorded as the maximum EAG amplitude relative to the baseline. If a notable decline in antennal responsiveness was observed during recording, the antenna was replaced with a fresh one from another individual.

### 2.4. Olfactory Behavioral Assay

Olfactory behavioral assays were conducted using a Y-tube olfactometer according to the method described by He et al. [31]. The Y-tube olfactometer consisted of an air sampler (QC-1B), a Y-tube (arm angle 75°, arm length 10 cm, inner diameter 1.5 cm), odor bottles, and a purification device, with all components connected by Teflon tubing. The assay was performed in a dark room at 25 °C, with the airflow rate set at 200 mL/min for each arm.

Each test compound was serially diluted with methanol to four concentrations: 0.1, 1, 10, and 100 mg/mL. For the treatment group, 200 μL of the diluted compound was applied to a cotton wick; for the control group, an equal volume of methanol was applied. After a 5 min odor equilibration period, a single cockroach was introduced into the base of the Y-tube and observed for 10 min. A cockroach was recorded as having chosen an odor if it moved beyond one-third of the arm length and remained there. Ten biological replicates (equal numbers of females and males) were tested for each compound, with each individual used only once. After each test, the orientation of the Y-tube arms was reversed to avoid positional bias, and the entire apparatus was cleaned with 95% ethanol and dried in an oven (DHG-9070A, Shanghai Jinghong Experimental Equipment Co., Ltd., Shanghai, China) at 37 °C.

The selection rate was calculated using the following formula:

$$Selection\;rate(\%)=\frac{\mathrm{Number}\;\mathrm{of}\;\mathrm{cockroaches}\;\mathrm{in}\;\mathrm{the}\;\mathrm{treatment}\;\mathrm{arm}}{Total\;number\;of\;cockroaches\;tested\;insect}\times 100$$

### 2.5. Statistical Analysis

Data were organized using Excel 2019. EAG response values of female and male adults to each compound were processed with EAGpro software(V2.0.2). EAG responses and sex-related differences were analyzed by Student’s *t*-test using SPSS 26.0. For olfactory behavioral assays in the Y-tube olfactometer, the Mann–Whitney U test (SPSS 26.0) was used to assess differences in choice preference between the treatment and control arms. This statistical analysis was performed based on raw choice outcomes from individual cockroach replicates (i.e., the count of individuals selecting either the treatment or control arm). The reported selection rate (%) is a descriptive summary calculated from these individual replicates and was not used as input for statistical testing. The significance threshold was set at *p* < 0.05. Graphs were created using OriginPro 2021.

## 3. Results

### 3.1. Identification of Volatile Organic Compounds in Cockroach Baits

In this study, HS-GC-MS was adopted to identify and characterize VOC profiles from 15 types of commercially available cockroach baits. Figure 1 displays total ion chromatograms of volatile constituents from each bait sample. Compound identification was confirmed via mass spectrum library matching, and the full VOC profiles of all baits are compiled in Table 1.

All volatile compounds detected across the 15 baits were sorted into eight major chemical classes: aromatics, hydrocarbons, ketones, furans, esters, aldehydes, alcohols, and organic acids, which are illustrated in the heatmap of Figure 2A. This heatmap quantifies the number of detectable compounds within each VOC class for every bait; darker coloration indicates more identified compounds.

The relative abundance of these eight compound classes differed significantly overall, with aldehydes contributing the largest proportion 19.3% (Figure 2B). In addition, volatile compositions differed greatly among distinct bait formulations. Figure S2 documents the peak area percentage of each volatile category for individual formulations. Taken together, these findings systematically elaborate the compositional regularities of volatile substances contained in commercial cockroach baits.

### 3.2. Electrophysiological Response of Key Volatiles in B. germanica

EAG recordings showed significant differences between male and female adults in response to four compounds, 2-methyl-3-buten-2-ol (*t*_28_ = 32.71, *p* = 3.40 × 10^−16^), 2-ethyl-1-hexanol (*t*_28_ = 2.63, *p* = 0.014), propylene glycol (*t*_28_ = 7.77, *p* = 6.00 × 10^−7^) and *p*-anisaldehyde (*t*_10_ = 4.91, *p* = 6.00 × 10^−4^) (Figure 3). In terms of electrophysiological activity, male adults showed the strongest antennal responses to 2-methyl-3-buten-2-ol, 2-ethyl-1-hexanol, hexanal, and propylene glycol, while female adults responded most strongly to hexanal and ethanol. For both hexanal and ethanol, clear EAG responses were observed in both sexes, and no significant sex differences were detected, although ethanol tended to elicit a slightly stronger response in females than in males.

### 3.3. Olfactory Behavioral Response of Key Volatiles in B. germanica

Y-tube olfactometer bioassays revealed distinct olfactory responses of cockroach adults to various volatile compounds at different concentrations. As illustrated in Figure 4, cockroach adults displayed significant attraction to multiple substances at specific concentrations: 2-methyl-3-buten-2-ol (0.1 mg/mL) (*U* = 90, *Z* = 3.487, *p* = 0.002), hexanal (1 mg/mL) (*U* = 80, *Z* = 2.615, *p* = 0.023), ethanol at 10 mg/mL (*U* = 80, *Z* = 2.615, *p* = 0.023) and 100 mg/mL (*U* = 80, *Z* = 2.615, *p* = 0.023)), 2-methylfuran at 1 mg/mL (*U* = 80, *Z* = 2.615, *p* = 0.023) and 10 mg/mL (*U* = 90, *Z* = 3.487, *p* = 0.002), propylene glycol (1 mg/mL) (*U* = 90, *Z* = 3.487, *p* = 0.002), ethyl butanoate (100 mg/mL) (*U* = 80, *Z* = 2.615, *p* = 0.023) and p-anisaldehyde (1 mg/mL) (U = 90, Z = 3.487, *p* = 0.002). Conversely, cockroach adults were repelled by 2-ethyl-1-hexanol (100 mg/mL) (*U* = 9, *Z* = −3.207, *p* = 0.004), acetic acid at 1 mg/mL (*U* = 20, *Z* = −2.615, *p* = 0.023) and 10 mg/mL (*U* = 10, *Z* = −3.487, *p* = 0.002), propanoic acid (100 mg/mL) (*U* = 20, *Z* = −2.615, *p* = 0.023), sorbic acid (100 mg/mL) (*U* = 0, *Z* = −4.359, *p* = 0.001), p-anisaldehyde (100 mg/mL) (*U* = 20, *Z* = −2.615, *p* = 0.023), γ-Nonalactone at 0.1 mg/mL (*U* = 20, *Z* = −2.615, *p* = 0.023). The sex-specific responses are presented separately in Figure S3.

## 4. Discussion

### 4.1. Screening of Potential Bioactive Volatiles in Cockroach Baits

Cockroach baits possess sophisticated formulation compositions. Beyond insecticidal active ingredients, these baits are formulated with multiple functional additives: excipients for shaping and texturing, preservatives for shelf-life prolongation, humectants for moisture retention, attractants for trapping target cockroaches, solvents for solubilizing bioactive components, and other miscellaneous adjuvants. During storage and field use, these diverse ingredients steadily emit volatile organic compounds (VOCs), jointly generating unique VOC fingerprints for different bait products [32].

Distinct variations could be observed across the 15 cockroach baits regarding detection frequency, distribution features and biological properties of diverse volatile compounds. Aldehydes occupied a relatively high proportion at 19.3% of total VOCs, which were mostly composed of C3–C8 short-chain aliphatic aldehydes such as 3-methylbutanal, 2-methylbutanal, 2-methylpropanal, propanal, butanal, pentanal and octanal. Most of these short-chain aliphatic aldehydes tended to occur in GB-5, GB-13 and GB-15 baits. p-Anisaldehyde was only found in GB-4, whereas hexanal could be detected in GB-3, GB-4, GB-7 and GB-8. Published research suggests p-anisaldehyde may exert bioactivity against *Frankliniella occidentalis* [18], and may serve as a repellent for *Amblyomma americanum* [33]. Hexanal has been shown to have potential attractant activity toward multiple insects, including *Dinoderus minutus* and *Holotrichia parallela* [14,19], these reports from other insect systems indicate that both compounds are behaviorally active and thus supported their selection for further testing in cockroaches.

Alcohols accounted for 16.1% of the total VOCs. Among them, ethanol was the most abundant alcohol detected, present in 12 out of the 15 baits. Other detected alcohols covered isopropyl alcohol, propylene glycol, 1-propanol, 2-ethyl-1-hexanol, and 2-methyl-3-buten-2-ol. Ethanol is a common metabolite of fermentation and has been reported that it may attract three species of *Ambrosia* beetles and two species of *Xylosandrus* [15,16]. In bait formulations, ethanol plays roles in improving stability and solubility, as well as adjusting bait consistency, as ethanol in the continuous phase has been shown to significantly retard sedimentation, creaming, and droplet coalescence, enabling relatively stable viscoelastic emulsions even without low-molecular-weight surfactants [34]. Even so, ethanol tends to volatilize quickly owing to its high volatility, which may limit its long-term retention inside baits. Isopropyl alcohol and propylene glycol mostly function as formulation adjuvants that possibly improve solubility and stability [35,36]. One study found that blending propylene glycol with terpenes in sugar-based attractants may strengthen attraction toward *Aedes aegypti* [21], Such results indicate that the inherent attractant capacity of propylene glycol has not been fully clarified, and its insect-attracting performance may vary with different formula compositions [37]. 2-Methyl-3-buten-2-ol and 2-ethyl-1-hexanol appear to have potential attracting activity against various insects. 2-Methyl-3-buten-2-ol acts as a key aggregation pheromone continuously emitted by male *Ips shangrila* [38] and has also been reported that it may attract *Hypsipyla grandella* [29] and *Monochamus galloprovincialis* when combined with other pheromones [20]. In contrast, 2-ethyl-1-hexanol presents a more complex picture: it has been shown to likely attract *Hippodamia variegata* [22] and *Ips typographus* [25], but also may exhibits repellent effects on *Megalurothrips usitatus* at high concentrations [31].

Organic acids accounted for 14.5% of the total VOCs. The identified organic acids mainly included acetic acid, propanoic acid, butanoic acid, sorbic acid, and benzoic acid, among which acetic acid was detectable in 8 of the 15 baits. Acetic acid, propanoic acid, and ethanol are typical metabolites produced by food fermentation. Existing studies have suggested that acetic acid may attract adult *Grapholita molesta* [24]. Short-chain organic acids such as propanoic acid and butanoic acid have been reported that they may be attractive to *Musca domestica* [26]. Notably, acetic acid and propanoic acid, together with ethanol, are typical metabolites of food fermentation. Considering *B. germanica* is an omnivorous insect, fermented food and its derived metabolites may be relatively appealing to this cockroach [39]. In contrast, the detected sorbic acid and benzoic acid were generally applied as preservatives in formulations [40].

Hydrocarbons accounted for 12.9% of the total VOCs, mainly including alkanes and alkenes. Alkanes were detected only in baits CB-13 and CB-14, while alkenes were detected in baits CB-7 and CB-13, D-limonene, one of the alkenes, was detected in bait CB-13. D-limonene has been shown not only to exhibit attractant activity toward *Bactrocera minax* and *Hypsipyla grandella* [29,30], but also to strongly attract *Trichogramma japonicum* [17] and *Bactrocera dorsalis* [23], suggesting that it may also have potential attractant effects on *B. germanica*.

Esters accounted for 9.7% of the total VOCs, with a distinct formulation-specific distribution pattern. Multiple ester compounds, including ethyl acetate, ethyl butyrate, and γ-nonalactone were detected in baits CB-4 and CB-8 which exhibited typical fruity and sweet odor characteristics during preparation and application. As for esters, previous studies have reported that ethyl acetate has potential attractant activity toward *Rhynchophorus ferrugineus* [27], while γ-nonalactone has been shown that it may exhibit repellent effects on *B. germanica* [41].

Aromatic compounds accounted for 8.1% of the total VOCs detected, mainly including styrene, benzaldehyde, and methylparaben. Styrene was detected in baits CB-4 and CB-10, while benzaldehyde was detected only in bait CB-8 and methylparaben was detected only in bait CB-3. Existing literature indicates styrene and benzaldehyde could attract the parasitic wasp *Aphidius colemani* [28]. Methylparaben is commonly used as a preservative to extend the shelf life of various foods [42], and few studies have so far documented its attraction toward insects.

Ketones occupied merely 6.5% of total VOCs, with four types (acetone, 2-butanone, cyclohexanone, 3-methyl-1,2-cyclopentanedione) found in CB-1, CB-2, CB-3, CB-5, CB-7, CB-8, CB-11, CB-12, CB-13 and CB-15. Up to now, published studies have rarely noted insect-attracting properties for these four ketones. Based on the formulation characteristics of the baits, acetone is speculated to function as a formulation adjuvant, acting as a solvent or solubilizer to improve the solubility and texture uniformity of the bait components [43].

Furans accounted for 4.8% of total VOCs. Only three furan compounds (furfural, 2-methylfuran, 2-pentylfuran) were detected at low levels in CB-1, CB-2, CB-3, CB-7, CB-8, CB-11, CB-14 and CB-15. Furan derivatives are commonly generated via non-enzymatic reactions during raw material heating: carbohydrate degradation and rearrangement from caramelization, amino acid thermal breakdown, and maillard reactions between reducing sugars and amino acids [44]. Hence, these furan compounds are hypothesized to form naturally as processing byproducts rather than intentional additives in bait recipes. Their bioactivity against *B. germanica* awaits additional verification.

Overall, aromatics, hydrocarbons, ketones and furans mostly exist as minor processing byproducts or formula additives, and their biological effects are largely unconfirmed. Esters and aldehydes appear capable of modulating insect behaviors, making them promising candidates for screening bioactive compounds targeting *B. germanica*. Alcohols and organic acids function dual roles: they may act as attractant cues originating from fermentation while also serving as functional excipients in bait formulas, which constitute the primary chemical basis for the trapping effect of baits. All volatile categories covered in this study encompass insect aggregation pheromones, plant volatiles, bait additives and fermentation metabolites. Multiple studies have implied these volatile substances may trigger attractant or repellent behaviors in diverse insect species.

### 4.2. Electrophysiological and Olfactory Behavioral Responses of Key Volatiles in B. germanica

Many VOCs detected in the present work have been documented to modulate insect behavioral responses [14,15,16,17,18,19,20,21,22,23,24,25,26,27,28,29,30]. Combining three screening indicators—the relative peak areas of major volatile peaks across all bait samples (Figure 1), detection frequency, and documented bioactivity from published literature against insects in general—we selected 14 candidate compounds for subsequent electroantennogram (EAG) and Y-tube olfactory behavioral tests. EAG combined with olfactory behavioral assays is a prevailing approach to assessing the bioactivity of volatile compounds acting on insects. EAG merely reflects insects’ peripheral perception of chemical stimuli instead of directly representing behavioral preference [45], which makes olfactory behavioral verification indispensable. By contrast, the combination of EAG and Y-tube olfactometer assays can more precisely characterize the behavioral orientation of insects toward odor cues [46]. In the present study, combined EAG and Y-tube olfactometer results demonstrated that 2-methyl-3-buten-2-ol and hexanal exerted strong and consistent electrophysiological activity as well as behavioral attraction toward *B. germanica*, implying their potential as core attractant ingredients for cockroach baits. In addition, D-limonene elicited relatively weak EAG responses but showed notable attraction in Y-tube olfactometer tests, whereas propylene glycol evoked strong EAG responses yet performed poorly in behavioral assays suggesting that the bioactivity of volatile compounds should be comprehensively evaluated by integrating both electrophysiological and behavioral evidence.

Sexual dimorphism in reproductive strategies and behavioral traits can induce intersexual variations in the morphology and distribution of antennal sensilla, olfactory physiological functions, and sensitivity to volatile compounds in insects. Pronounced sex-based differences in EAG responses have been documented in *Blattella asahinai* [47], *Lycorma delicatula* [48] and *Theocolax elegans* [49]. In the present study, all 14 tested volatile compounds triggered obvious EAG responses in *B. germanica*, among which four compounds presented significant intersexual differences. Female adults were more sensitive to *p*-anisaldehyde, whereas males showed stronger electrophysiological responses to 2-methyl-3-buten-2-ol, 2-ethyl-1-hexanol, and propylene glycol. Hexanal elicited similarly high EAG responses in both sexes. In addition, olfactory behavioral responses to the 14 volatile compounds differed between female and male *B. germanica* (Figure S3). These findings suggest the presence of olfactory sexual divergence in *B. germanica* and may provide a useful reference for future investigations into sex-related olfactory differences in insects.

Insect behavioral responses to odorants typically exhibit a typical concentration-dependent pattern, which has been well reported in *Drosophila melanogaster* and *Phlebotomus papatasi* [50,51]. Likewise, palmitic acid exerts concentration-dependent effects on *B. germanica*, showing attraction at low concentrations and repellency at high concentrations [52]. In the olfactory behavioral experiments, the effect of p-anisaldehyde on the test cockroaches was similar, at a lower concentration of 1 mg/mL, it exhibited attractant activity, but at a high concentration of 100 mg/mL, it showed repellent effects on cockroaches. Meanwhile, the experiment found that acids tended to repel cockroaches at higher concentrations. For example, cockroaches displayed avoidance behavior toward acetic acid, propionic acid, and sorbic acid at the high concentration of 100 mg/mL. In addition, ethanol at concentrations of 10 mg/mL and 100 mg/mL, and propylene glycol at 1 mg/mL, both showed attractant effects on cockroaches, suggesting that short-chain alcohol compounds may have attractant potential for cockroaches. γ-nonalactone exhibited repellent effects on cockroaches at a low concentration of 0.1 mg/mL, which is consistent with findings described in the literature that γ-nonalactone may repel *B. germanica* at certain vapor-phase concentrations. As for 2-methylfuran, it showed attractant effects at 1 mg/mL and 10 mg/mL; however, because its EAG responses showed relatively small electrical signals in stimulating cockroach antennae, further research is needed to determine whether it can serve as a core attractant in bait formulations. Above all, our behavioral assays revealed that 2-methyl-3-buten-2-ol and hexanal maintained stable attractive effects on *B. germanica* at low concentrations and elicited relatively high EAG responses, which provide valuable references for the development of novel cockroach attractants. Moreover, this study only evaluated the biological activity of single compounds; the potential synergistic effects of volatile compound mixtures remain worthy of further investigation in future work.

## 5. Conclusions

This study adopted HS-GC-MS to characterize VOCs of commercial cockroach baits. EAG detection and Y-tube olfactory bioassays were further applied to explore olfactory physiological and behavioral responses of adult *B. germanica* to the acquired volatile compounds. Combining chemical identification and functional verification data, aldehydes, hydrocarbons, alcohols and organic acids are speculated to be the major volatile groups that may drive olfactory attraction of the baits toward cockroaches. Notably, two compounds, namely 2-methyl-3-buten-2-ol and hexanal, tended to trigger intense EAG signals and steady behavioral attraction in relatively low tested concentration treatments. These compounds may play key roles in guiding the olfactory preference of *B. germanica* to commercial cockroach baits. The present work successfully distinguished bioactive volatiles from commercial cockroach baits and pinpointed key odor attractants from complicated volatile blends. The relevant results may supplement research on the molecular olfactory mechanism of bait attraction and deepen knowledge regarding food-borne semiochemicals linked to *B. germanica*. Collectively, the data offer preliminary clues to clarify the chemical basis accounting for the attractive capacity of commonly used cockroach baits. The screened pivotal volatile substances may furnish theoretical evidence and technical guidance for subsequent formula optimization, removal of useless components, and exploitation of novel specific attractants for *B. germanica* control. In addition, this research may lay tentative theoretical groundwork for designing environmentally friendly cockroach prevention tactics relying on olfactory and behavioral regulation.

## Figures and Tables

**Figure 1:**
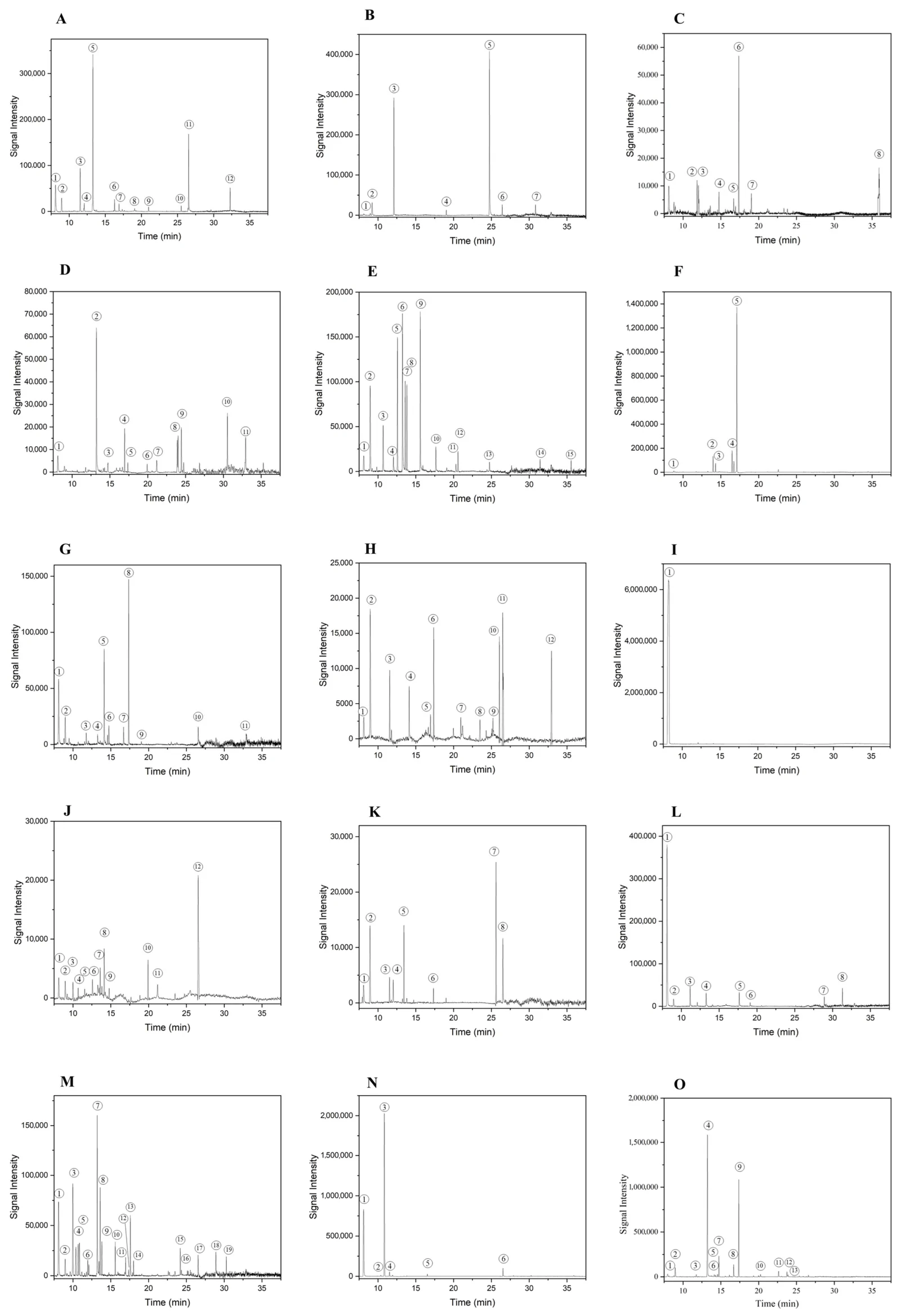
HS-GC-MS chromatograms of 15 cockroach baits. The 15 baits were labeled as (**A**): Cockroach bait 1, CB-1; (**B**): Cockroach bait 2, CB-2; (**C**): Cockroach bait 3, CB-3; (**D**): Cockroach bait 4, CB-4, (**E**): Cockroach bait 5, CB-5, (**F**): Cockroach bait 6, CB-6; (**G**): Cockroach bait 7, CB-7; (**H**): Cockroach bait 8, CB-8; (**I**): Cockroach bait 9, CB-9; (**J**): Cockroach bait 10, CB-10; (**K**): Cockroach bait 11, CB-11; (**L**): Cockroach bait 12, CB-12; (**M**): Cockroach bait 13, CB-13; (**N**): Cockroach bait 14, CB-14; (**O**): Cockroach bait 15, CB-15. The peaks labeled in the chromatograms correspond to the peak numbers listed in Table 1. Among these labelled peaks, A ⑫; B⑥⑦; C ④⑤; D ③⑦; E ⑭⑮; G ③⑪; H ④⑦; J ②③④⑤⑥⑦⑧⑨⑪; K ⑤⑥ and M⑲ were excluded from the table, as their NIST 20 mass spectral library match scores fall below 80, rendering them unreliable for qualitative analysis.

**Figure 2:**
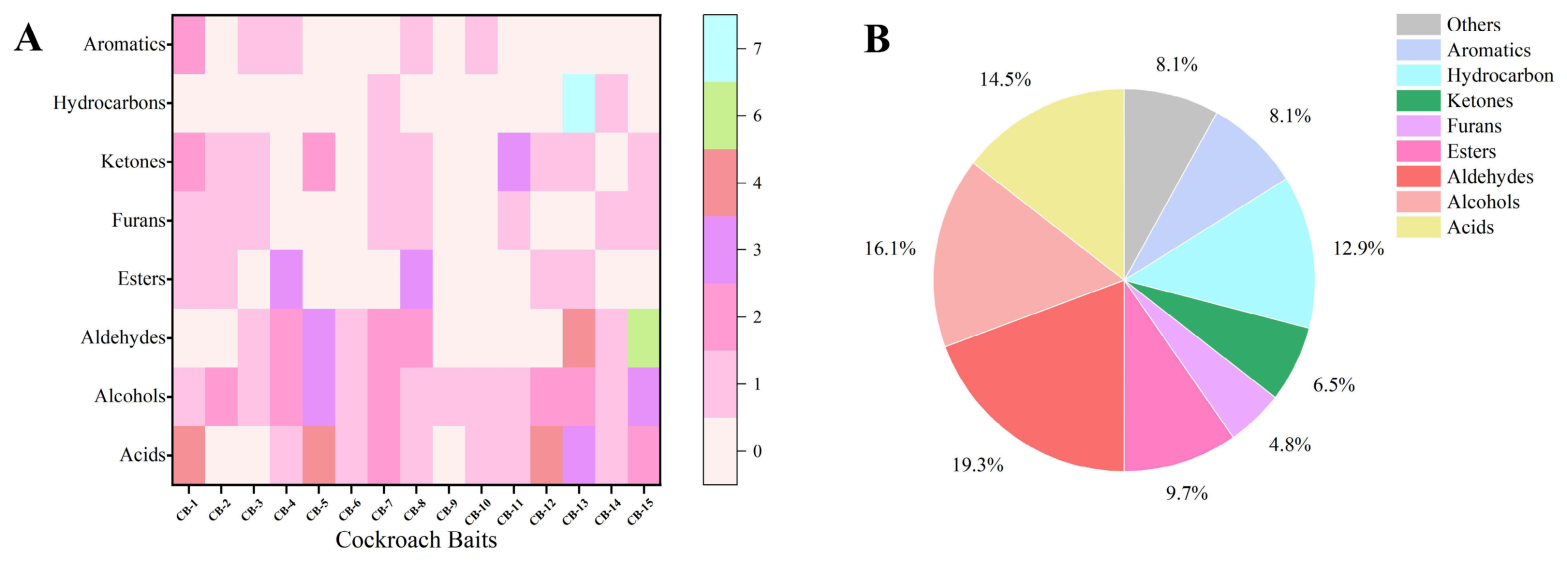
Heatmap of VOCs categories and their proportional distribution in 15 commercial cockroach baits. (**A**): Heatmap of VOCs category distribution across 15 cockroach baits, the color intensity of each cell represents the number of distinct volatile compounds. (**B**): Pie chart showing the proportion of each VOCs category among all detected volatiles.

**Figure 3:**
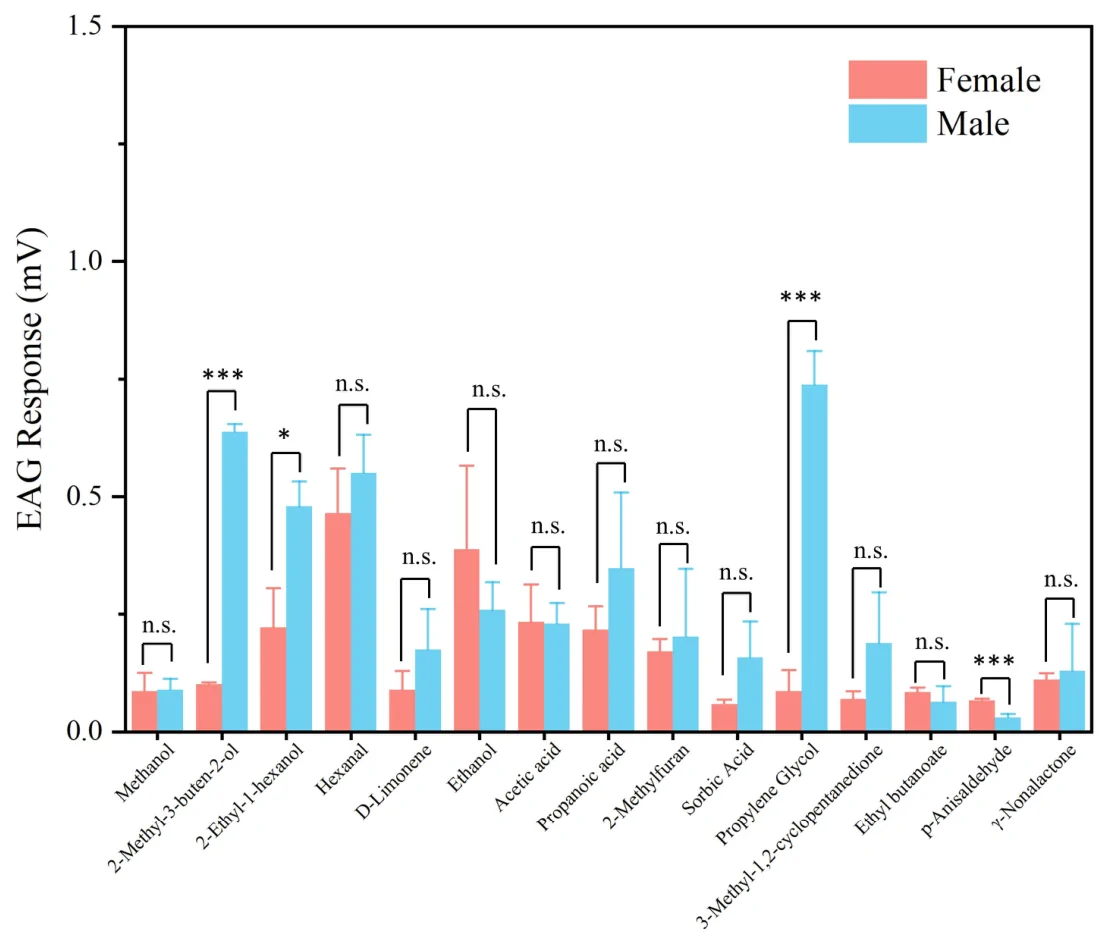
Electroantennogram responses of *B. germanica* to 14 volatile compounds. Data were mean ± SE. * *p* < 0.05, *** *p* < 0.001, n.s. indicates not significant.

**Figure 4:**
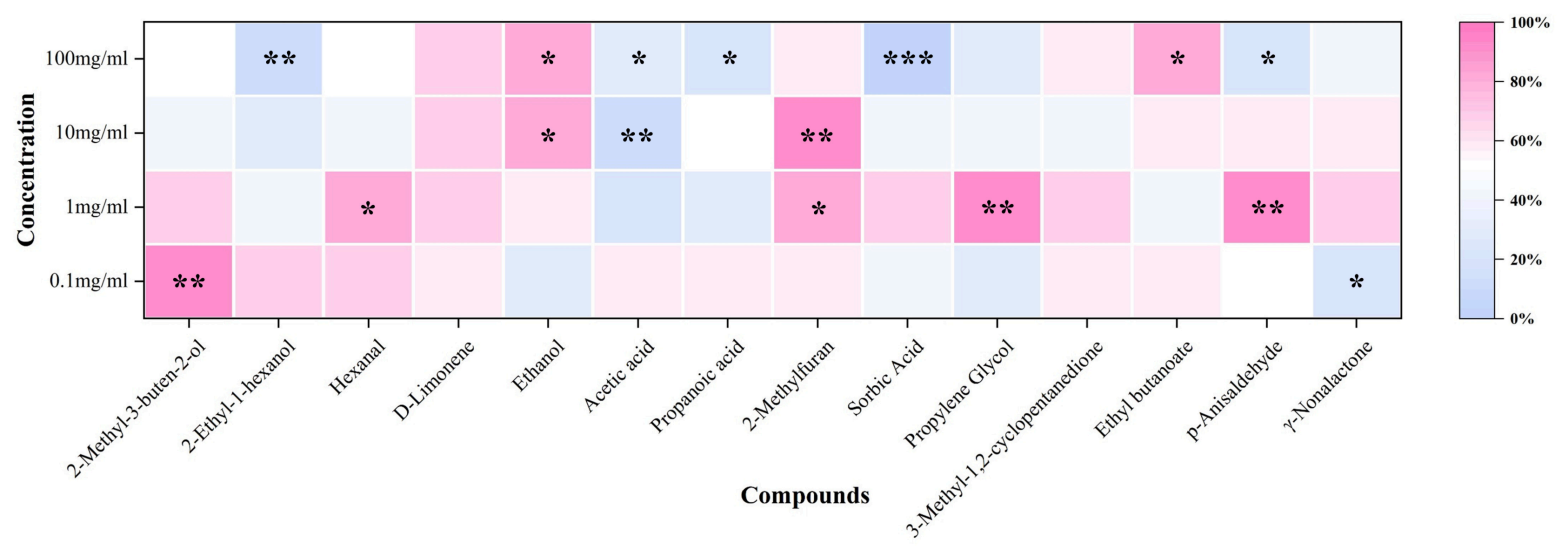
Olfactory behavioral responses of *B. germanica* to 14 volatile compounds. Color of each cell represents the selection rate of cockroaches responding to the corresponding compound, with red indicating positive attraction and blue indicating avoidance; * *p* < 0.05, ** *p* < 0.01, *** *p* < 0.001.

**Table 1:** Volatile compounds identified in 15 cockroach baits by HS-GC-MS.

Bait Code	No.	RT (min)	Compound Name	CAS	Peak Area	Relative Percentage
CB-1	1	8.1	Ethanol	64-17-5	403,266.3 ± 3330.7	18.0%
	2	9.0	Acetone	67-64-1	176,901.3 ± 6708.1	7.9%
	3	11.5	2-Methylfuran	534-22-5	377,193.8 ± 22,253.5	16.9%
	4	12.1	Ethyl acetate	141-78-6	66,809.3 ± 3614.8	3.0%
	5	13.3	Acetic acid	64-19-7	761,924.5 ± 69,175.4	34.1%
	6	16.3	Toluene	108-88-3	67,522.0 ± 2992.2	3.0%
	7	16.9	2-Methylpropanoic acid	79-31-2	39,462.5 ± 4259.1	1.8%
	8	19.1	3-Methylbutanoic acid	503-74-2	12,929.2 ± 1836.8	0.6%
	9	21.0	Cyclohexanone	108-94-1	32,558.8 ± 769.3	1.5%
	10	25.5	Benzyl ethyl ether	539-30-0	30,671.5 ± 1348.4	1.4%
	11	26.5	Sorbic acid	110-44-1	266,695.8 ± 77,603.6	11.9%
CB-2	1	9.0	Acetone	67-64-1	25,879.5 ± 1657.8	1.1%
	2	9.2	Isopropyl alcohol	67-63-0	168,638.5 ± 4947.8	7.1%
	3	12.1	Ethyl acetate	141-78-6	830,798.8 ± 60,758.4	35.0%
	4	19.1	Furfural	98-01-1	41,885.0 ± 2155.4	1.8%
	5	24.8	2-Ethyl-1-hexanol	104-76-7	1,306,172.3 ± 43,569.4	55.0%
CB-3	1	8.1	Ethanol	64-17-5	28,091.3 ± 1468.8	5.9%
	2	11.9	Nitromethane	75-52-5	37,135.0 ± 2340.2	7.8%
	3	12.0	2-Butanone	78-93-3	30,404.0 ± 1538.2	6.4%
	6	17.4	Hexanal	66-25-1	150,602.8 ± 5947.9	31.5%
	7	19.0	Furfural	98-01-1	21,741.3 ± 998.6	4.5%
	8	35.9	Methylparaben	99-76-3	210,574.0 ± 80,903.9	44.0%
CB-4	1	8.1	Ethanol	64-17-5	26,163.0 ± 2728.1	4.4%
	2	13.2	Acetic acid	64-19-7	220,022.8 ± 7839.8	37.0%
	4	16.9	Ethyl butanoate	105-54-4	71,927.3 ± 8751.4	12.1%
	5	17.4	Hexanal	66-25-1	15,317.0 ± 1410.3	2.6%
	6	19.9	Styrene	100-42-5	12,102.5 ± 2028.3	2.0%
	8	24.4	1-Methoxy-2-propyl acetate	20324-32-7	76,403.0 ± 1449.8	12.8%
	9	24.8	2-Ethyl-1-hexanol	104-76-7	16,094.5 ± 2374.2	2.7%
	10	30.5	p-Anisaldehyde	123-11-5	75,792.8 ± 1210.5	12.7%
	11	32.9	γ-Nonalactone	104-61-0	81,510.3 ± 5324.0	13.7%
CB-5	1	8.1	Ethanol	64-17-5	102,703.5 ± 2923.6	3.2%
	2	9.0	Acetone	67-64-1	522,111.0 ± 16,169.6	16.3%
	3	10.7	2-Methylpropanal	78-84-2	244,010.0 ± 12,948.1	7.6%
	4	12.0	2-Butanone	78-93-3	68,106.3 ± 2207.8	2.1%
	5	12.6	2-Methyl-3-buten-2-ol	115-18-4	508,775.3 ± 20,355.3	15.9%
	6	13.2	Acetic acid	64-19-7	503,411.0 ± 53,140.8	15.7%
	7	13.6	3-Methylbutanal	590-86-3	320,631.3 ± 18,344.5	10.0%
	8	13.8	2-Methylbutanal	96-17-3	309,028.3 ± 18,977.9	9.7%
	9	15.6	Propanoic acid	79-09-4	404,757.8 ± 34,611.7	12.6%
	10	17.7	Butanoic acid	107-92-6	72,275.3 ± 6170.5	2.3%
	11	20.3	Propylmalonic acid	616-62-6	25,344.8 ± 5052.1	0.8%
	12	20.6	2,6-Dimethyl-pyrazine	108-50-9	82,785.0 ± 2511.6	2.6%
	13	24.8	2-Ethyl-1-hexanol	104-76-7	36,968.3 ± 496.1	1.2%
CB-6	1	8.8	Propanal	123-38-6	61,881.5 ± 1746.0	1.0%
	2	13.2	Acetic acid	64-19-7	20,281.5 ± 11,719.2	0.3%
	3	14.0	2-Methyl-1,3-dioxane	626-68-6	363,907.5 ± 4230.6	5.9%
	4	16.5	2-Ethyl-4-methyl-1,3-dioxolane	4359-46-0	472,830.0 ± 10,940.5	7.7%
	5	17.1	Propylene glycol	57-55-6	5,213,635.3 ± 1,964,287.4	85.0%
CB-7	1	8.1	Ethanol	64-17-5	287,993.0 ± 49,781.1	25.9%
	2	9.0	Acetone	67-64-1	87,434.8 ± 19,956.6	7.9%
	4	13.3	Acetic acid	64-19-7	27,544.5 ± 1329.9	2.5%
	5	14.1	1-Butanol	71-36-3	235,920.8 ± 4872.4	21.2%
	6	14.7	Pentanal	110-62-3	42,338.5 ± 1607.4	3.8%
	7	16.7	1-Pentene	109-67-1	38,992.5 ± 1251.7	3.5%
	8	17.4	Hexanal	66-25-1	335,303.0 ± 17,864.5	30.1%
	9	19.0	Furfural	98-01-1	10,186.5 ± 613.5	0.9%
	10	26.6	Sorbic acid	110-44-1	46,568.5 ± 4595.4	4.2%
CB-8	1	8.1	Ethanol	64-17-5	11,160.8 ± 2742.8	2.7%
	2	9.0	Acetone	67-64-1	99,596.5 ± 4141.5	24.3%
	3	11.5	2-Methylfuran	534-22-5	34,428.5 ± 4049.7	8.4%
	5	17.0	Ethyl butanoate	105-54-4	9417.5 ± 362.4	2.3%
	6	17.4	Hexanal	66-25-1	46,737.3 ± 714.8	11.4%
	8	23.5	Benzaldehyde	100-52-7	10,458.8 ± 1281.0	2.5%
	9	25.2	1,4-Diethyl- benzene	105-05-5	8159.8 ± 952.5	2.0%
	10	26.1	Ethyl levulinate	539-88-8	36,975.8 ± 3763.3	9.0%
	11	26.6	Sorbic acid	110-44-1	70,824.8 ± 2364.2	17.2%
	12	32.9	γ-Nonalactone	104-61-0	82,873.3 ± 1233.2	20.2%
CB-9	1	8.1	Ethanol	64-17-5	53,118,853.8 ± 1,057,550.6	100%
CB-10	1	8.1	Ethanol	64-17-5	8622.8 ± 1957.2	10.9%
	10	19.9	Styrene	100-42-5	22,115.0 ± 999.8	27.9%
	12	26.6	Sorbic acid	110-44-1	48,429.0 ± 2234.2	61.2%
CB-11	1	8.1	Ethanol	64-17-5	22,587.3 ± 881.9	8.0%
	2	9.0	Acetone	67-64-1	77,650.8 ± 1357.4	27.4%
	3	11.5	2-Methylfuran	534-22-5	17,392.8 ± 512.4	6.1%
	4	12.0	2-Butanone	78-93-3	14,932.0 ± 344.2	5.3%
	7	25.6	3-Methyl-1,2-cyclopentanedione	765-70-8	102,400.5 ± 15,568.5	36.1%
	8	26.5	Sorbic acid	110-44-1	48,641.5 ± 10,372.2	17.2%
CB-12	1	8.1	Ethanol	64-17-5	2,477,803.3 ± 220,820.1	78.7%
	2	9.0	Acetone	67-64-1	79,645.0 ± 12,151.8	2.5%
	3	11.2	1-Propanol	71-23-8	186,548.5 ± 11,231.2	5.9%
	4	13.3	Acetic acid	64-19-7	119,269.3 ± 41,770.8	3.8%
	5	17.7	Butanoic acid	107-92-6	97,579.5 ± 10,526.6	3.1%
	6	19.1	3-Methylbutanoic acid	503-74-2	21,985.5 ± 7467.6	0.7%
	7	28.9	Benzoic acid	65-85-0	32,792.8 ± 12,152.6	1.0%
	8	31.3	Triacetin	102-76-1	134,389.8 ± 12,506.5	4.3%
CB-13	1	8.1	Ethanol	64-17-5	424,283.3 ± 14,554.2	16.9%
	2	9.0	Acetone	67-64-1	49,268.3 ± 12,691.0	2.0%
	3	10.0	2-Methyl-2-propanol	75-65-0	384,793.5 ± 18,165.0	15.3%
	4	10.4	3-Methylpentane	96-14-0	103,360.5 ± 17,586.0	4.1%
	5	10.7	2-Methylpropanal	78-84-2	122,451.0 ± 6881.2	4.9%
	6	12.1	Ethyl acetate	141-78-6	33,872.0 ± 2194.3	1.3%
	7	13.2	Acetic acid	64-19-7	464,194.8 ± 3422.9	18.4%
	8	13.6	3-Methylbutanal	590-86-3	234,006.8 ± 11,570.8	9.3%
	9	13.8	2-Methylbutanal	96-17-3	89,671.3 ± 5564.1	3.6%
	10	15.6	4-Methylheptane	589-53-7	76,745.5 ± 3667.3	3.0%
	11	17.0	3-Ethyl-3-methylheptadecane	714275-12-4	54,026.0 ± 2291.8	2.1%
	12	17.4	Hexanal	66-25-1	16,860.8 ± 542.2	0.7%
	13	17.6	2,4-Dimethyl-1-heptene	19549-87-2	153,643.3 ± 10,371.4	6.1%
	14	18.0	4-Methyloctane	2216-34-4	38,675.5 ± 2795.1	1.5%
	15	24.2	D-Limonene	138-86-3	69,485.3 ± 3913.7	2.8%
	16	24.3	3,3-Dimethylhexane	563-16-6	14,872.0 ± 1698.3	0.6%
	17	26.5	Sorbic acid	110-44-1	150,242.0 ± 40,538.8	6.0%
	18	28.9	Benzoic acid	65-85-0	36,674.8 ± 10,913.1	1.5%
CB-14	1	8.1	Ethanol	64-17-5	4,438,715.0 ± 258,159.6	41.7%
	2	10.4	3-Methylpentane	96-14-0	41,953.5 ± 4619.2	0.4%
	3	10.9	2-Ethyl-oxetane	4737-47-7	5,742,131.8 ± 674,827.6	53.9%
	4	11.5	2-Methylfuran	534-22-5	198,858.8 ± 7034.9	1.9%
	5	16.5	2-Pentenal	764-39-6	77,562.5 ± 1402.8	0.7%
	6	26.5	Sorbic acid	110-44-1	147,636.5 ± 46,434.6	1.4%
CB-15	1	8.8	Propanal	123-38-6	32,403.5 ± 18,729.0	0.3%
	2	9.0	Acetone	67-64-1	561,214.8 ± 26,475.1	4.8%
	3	11.8	Butanal	123-72-8	90,823.0 ± 5204.1	0.8%
	4	13.2	Acetic acid	64-19-7	6,446,039.8 ± 660,461.5	55.4%
	5	14.1	1-Butanol	71-36-3	53,235.8 ± 18,224.0	0.5%
	6	14.6	1-Penten-3-ol	616-25-1	83,168.8 ± 3524.2	0.7%
	7	14.8	Pentanal	110-62-3	579,367.5 ± 23,364.8	5.0%
	8	16.7	1-Pentanol	71-41-0	448,697.5 ± 26,391.0	3.9%
	9	17.4	Hexanal	66-25-1	2,766,121.8 ± 95,937.4	23.8%
	10	20.3	Heptanal	111-71-7	89,998.8 ± 1957.5	0.8%
	11	22.6	2-Pentylfuran	3777-69-3	178,972.5 ± 23,500.5	1.5%
	12	23.8	Hexanoic acid	142-62-1	255,607.8 ± 42,206.3	2.2%
	13	23.9	Octanal	124-13-0	56,364.0 ± 18,849.9	0.5%

Note: Peak area data are presented as mean ± SE. The relative percentage of each compound was calculated as the peak area of the individual compound divided by the total peak area of all VOCs with a match score > 80.

## Data Availability

All data from this study are available in this paper and the associated papers.

## Supplementary Materials

The following supporting information can be downloaded at: https://www.mdpi.com/article/10.3390/insects17101010/s1, Table S1: Basic information of the 15 commercially available cockroach baits; Figure S1: Total ion chromatogram of the 14 key volatile organic compound standards; Figure S2: Proportion of peak areas of each volatile category in each cockroach bait formulation (labeled as A–O, corresponding to Cockroach Bait 1–15, CB-1 to CB-15) among all detected volatiles by HS-GC-MS. Figure S3: Sex-specific olfactory behavioral responses of *Blattella germanica* to 14 volatile compounds.
