# Supplementary material for: Characterization and Behavioral Responses of Volatile Organic Compounds in Commercial Cockroach Baits
Source: Insects. 2026 Sep 30;17(10):1010. doi: 10.3390/insects17101010 (PMC13705419; doi:10.3390/insects17101010)
Supplement: Supplementary file 1 [file insects-17-01010-s001.zip › insects-4519436-supplementary.pdf]

Characterization and behavioral responses of volatile organic compound in commercial cockroach baits

Kejia Chen+, Bin Sun+, Hongyan Lyu, Xinhong Xu, Lu Jiang, Xiaomeng Ren, Huiling Hao*, Yunchuan He*

Chinese People's Liberation Army Naval Medical Center, Shanghai 200433, China

*Corresponding author: he_yunchuan@126.com, hhl18916873229@163.com

+These authors contributed equally to this work.

Table S1 Basic information of the 15 commercially available cockroach baits

Bait code	Active ingredient	Concentration	Manufacturer
1	Indoxacarb	0.05%	Company 1
2	Imidacloprid	2.15%	Company 2
3	Cockroach virus	6000 PIB/g	Company 3
4	Indoxacarb	0.5%	Company 2
5	Dinotefuran	0.5%	Company 4
6	Hydramethylnon	2.0%	Company 4
7	Imidacloprid	2.5%	Company 5
8	Dinotefuran	0.2%	Company 6
9	Hydramethylnon	2.0%	Company 7
10	Imidacloprid	2.5%	Company 8
11	Imidacloprid	2.5%	Company 9
12	Dinotefuran	0.5%	Company 10
13	Imidacloprid	2.5%	Company 10
14	Imidacloprid	2.5%	Company 11
15	Indoxacarb	0.5%	Company 12

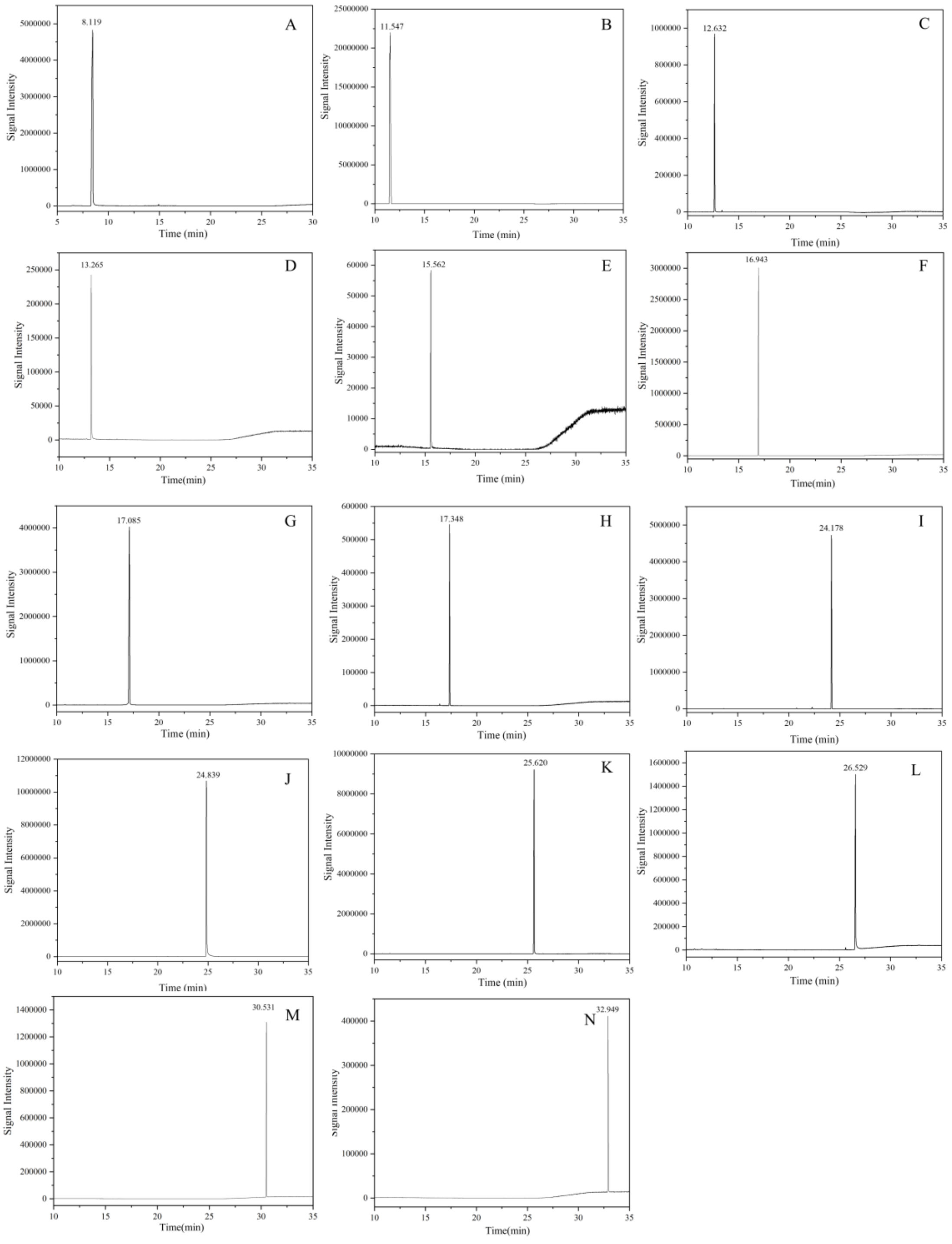

Figure S1 Total ion chromatogram of the 14 key volatile organic compound standards, A: Ethanol; B: 2-Methylfuran; C: 2-methyl-3-Buten-2-ol; D: Acetic acid; E: Propanoic acid; F: Ethyl butanoate; G: Propylene glycol; H: Hexanal; I: D-Limonene; J: 1-Hexanol, 2-ethyl-; K: 1,2-Cyclopentanedione, 3-methyl-; L: Sorbic acid; M: p-Anisaldehyde; N: γ-Nonalactone.

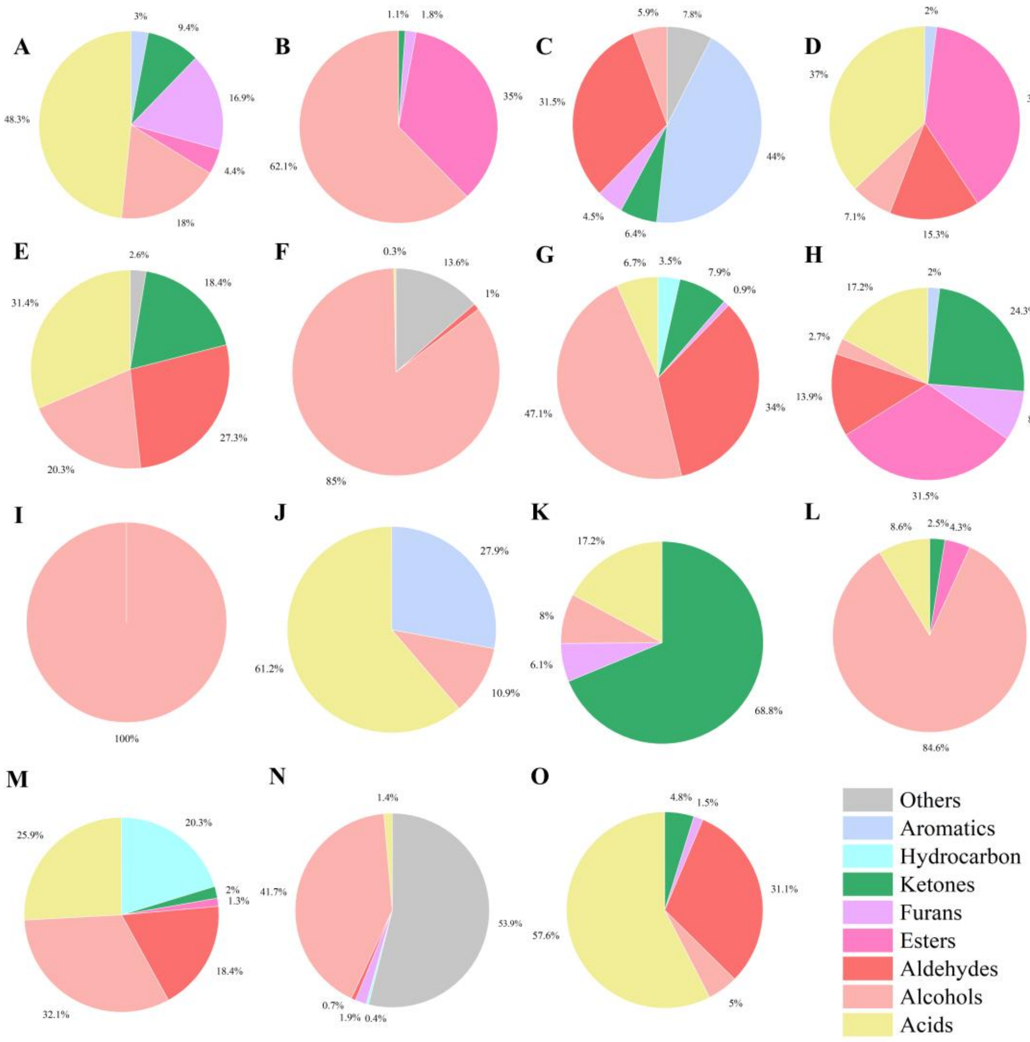

Figure S2 Proportion of peak areas of each volatile category in each cockroach bait formulation (labeled as A–O, corresponding to Bait 1–15, CB-1 to CB-15) among all detected volatiles by HS-GC-MS.

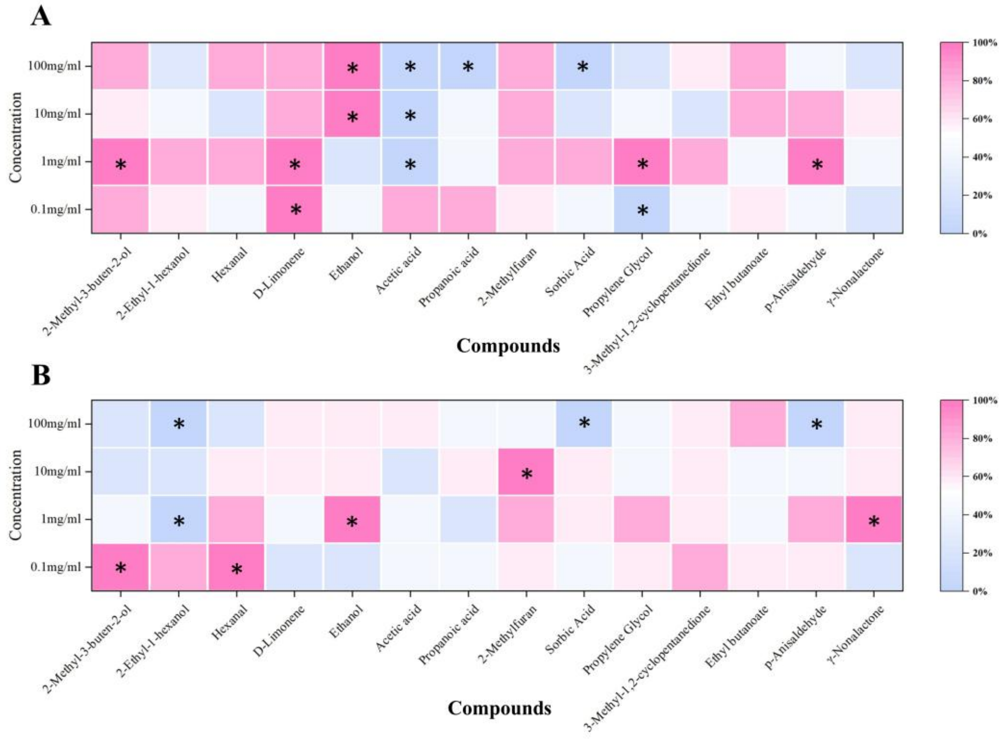

Figure S3 Sex-specific olfactory behavioral responses of *Blattella germanica* to 14 volatile compounds. A: Female adults; B: Male adults. Color of each cell represents the selection rate (%) of cockroaches responding to the corresponding compound, with red indicating selection rate > 50% and blue indicating selection rate < 50%. Asterisks indicate significant differences between sexes (*$P < 0.05$).
